# Safety Profile of Thiopurines in Crohn Disease: Analysis of 893 Patient-Years Follow-Up in a Southern China Cohort

**DOI:** 10.1097/MD.0000000000001513

**Published:** 2015-10-16

**Authors:** Yun Qiu, Ren Mao, Sheng-hong Zhang, Man-ying Li, Jing Guo, Bai-li Chen, Yao He, Zhi-rong Zeng, Min-hu Chen

**Affiliations:** From the Department of Gastroenterology, First Affiliated Hospital of Sun Yat-Sen University, Guangzhou, People's Republic of China.

## Abstract

Thiopurines have been associated with both clinical improvement and mucosal healing in treating Crohn disease (CD). Unfortunately, the high rate of adverse events (AEs) leading to drug withdrawal represents a major limitation in the use of these drugs.

To evaluate the safety of thiopurines in patients with CD. To identify predictive factors associated with the development of thiopurine-induced AEs and withdrawal.

This longitudinal cohort study examined patients from a university-based IBD referral center. Time-to-event analysis was performed with the Kaplan–Meier curve. Cox regression analysis was performed to identify potential predictive factors of AEs.

Two hundred sixty-seven CD patients on thiopurines were included. A total of 143 AEs occurred at a median of 7.4 months (interquartile range, 3.7–15.3 months) after starting treatment. The cumulative incidence of AEs was 26%, with an annual risk of 4.3% per patient-year of treatment. The most frequent was leucopenia (41/267, 15.36%), followed by infections (29/267, 10.86%). Independent factors predictive of leucopenia were lower baseline hemoglobin (hazard ratio (HR), 0.34; 95% confidence interval (CI) 0.18–0.67) and the concomitant use of 5-aminosalicylic acid (HR, 3.05; 95% CI 1.44–8.76). Of the 28.44% (76/267) CD patients discontinued therapy, 14.61% due to AEs. A lower body mass index, the presence of extraintestinal manifestation, and the incidence of leucopenia independently predicted thiopurine withdrawal. In total, 37.5% of these patients restarted thiopurines and 52.3% of them had AEs again.

About a quarter of patients on thiopurine therapy had AEs during follow-up and 1 of 7 patients had to discontinue thiopurines due to AEs.

## INTRODUCTION

The efficacy of thiopurine immunomodulators azathioprine (AZA) and its metabolite 6-mercaptopurine (6-MP) for maintenance of remission in patients with Crohn disease (CD), albeit modest, is well established,^1^ and the benefit/risk ratio favors their use in most cases of disabling CD.^2^ From recent data, it appears that thiopurines use is able to change the natural history of disease for reason of decreasing the surgery rate in CD.^3^ Furthermore, thiopurine therapy impacts positively health-related quality of life,^4^ and, thus, thiopurine has long become the most common immunosuppressive therapies used in CD.

Unfortunately, thiopurine therapy is frequently hampered by adverse events (AEs) that occur in approximately 15% to 40% of patients leading to dose reduction or drug withdrawal.^5^ Chaparro et al using a Spanish database demonstrated as many as 1 of 4 patients with inflammatory bowel disease (IBD) on thiopurine therapy had AEs which lead to 17% treatment cessation.^6^ However, the study comprised a mixed cohort that included patients with CD, ulcerative colitis, and unclassified colitis.

Since response to and toxicity of any pharmacological treatment are partly based on the genetic background of the population, we retrospectively assessed the frequency and type of side effects in an IBD population of Southeastern China followed at our IBD center. It also demonstrated that AEs can occur at any time and are frequently unpredictable,^7^ another goal of this study was to determine the surrogate predictors for AEs and drug withdrawal.

## METHODS

### Patients and Design

This was a longitudinal cohort study. All consecutive patients with an established diagnosis of CD according to the criteria of Lennard-Jones^8^ who received AZA/6-MP treatment at the Gastroenterology Outpatient Clinic of the First Affiliated Hospital of Sun Yat-sen University between 2000 and 2013 were included in this study. All patient treatments were supervised by a senior gastroenterologist (CMH) since 2003.

AZA/6-MP was given to all CD patients who fulfilled a set of criteria: moderate to severely active ileocecal or colonic CD;^9^ clinical factors that suggested a poor prognosis (diagnosis before 40 years of age, perianal disease, extensive involvement of the colon, or deep ulceration); steroid dependency or extensive small bowel or esophageal/gastroduodenal involvement.

Exclusion criteria of this study: age younger than 18 years; patients with severe comorbidity, documented infection, renal or liver failure, or other contraindication to thiopurines according to labeling recommendations; patients with a minimal follow-up of 6 months.

The study protocol was approved by the Clinical Research Ethics Committee of The First Affiliated Hospital of Sun Yat-sen University.

### Treatment Schedules: Dosing and Duration

According to the major available guidelines^10–12^ AZA dose was targeted at 2.0 to 2.5 mg/kg body weight and 6-MP at 1.0 to 1.5 mg/kg body weight. Since thiopurine methyltransferase (TPMT) testing is not available routinely before 2005, drug dose was started with 1 mg/kg daily for AZA (Imuran, GlaxoSmithKline, Solna, Sweden) or 0.5 mg/kg daily for 6- MP (Purinethol, GlaxoSmithKline) in the first week, and then gradually increase it up to the target dose by regular monitoring the 6-thioguanine nucleotides (6-TGNs) concentrations to achieve the therapeutic window of 250–400 pmol/8 × 10^8^ erythrocyte.

Thiopurine metabolite concentrations were usually measured 8 to 12 weeks after initiation of thiopurines. We decided to use the thiopurine metabolite concentrations after 3 months because steady-state 6-TGN concentrations may be anticipated following 2 months of use.^13^

### Clinical Follow-Up

The clinical data documented in the medical files and the IBD register of the patients were reassessed by 2 experienced gastroenterologists (B-LC, YH). A predetermined data sheet was used to collect information such as symptoms of the disease prior to and during the thiopurines medication, thiopurines initiation dates and dosage, and comedication. The laboratory values of hematology, immunology, chemistry, and microbiology, the incidence of AEs, subsequent medication adjustment and response, the cessation of thiopurines including reasons were collected at successive visits.

### Toxicity of Thiopurines

The definition for AE was adopted by Jharap et al, that is, any new symptom or sign, any significant laboratory abnormality, or worsening of a preexisting condition or abnormality that occurred after initiation of thiopurines was considered an AE.^5^ AEs were further subdivided into myelotoxicity, pancreatitis, hepatotoxicity, gastrointestinal complaints, infection, influenza-like symptoms, arthralgia, and others. The definitions are as follows: leucopenia: total white cell count less than 3.5 × 10^9^/L ^3,14–16^; neutropenia: neutrophil count <1.5 × 10^9^/L in 2 days continuously, and recovered in the next 1 to 2 weeks after cessation of thiopurine; myelotoxicity: the presence of leucopenia, neutropenia, thrombocytopenia (<100 × 10^9^/L), or a hemoglobin level below the lower reference limit; pancreatitis: upper abdominal pain and a 2-fold increase of amylase or lipase activities above the upper reference interval limit; and hepatotoxicity: 2.5-fold increase of alanine aminotransferase and aspartate aminotransferase activities above the upper limit of normal and/or increased bilirubin concentration >51.3 μmol/L (3 mg/dL).

Reasons for discontinuation of thiopurines were subclassified into AEs, clinical refractoriness or undergone operation, sustained clinical remission, safety concerns, or noncompliance.

### Statistical Analysis

Demographic and clinical parameters were compiled and summary statistics calculated. Data were described using medians with interquartile range (IQR) for continuous data and percentages for discrete data. For statistical analysis Fisher exact test and *χ*^2^ tests were used to compare the nonparametric categorical data between groups and analysis of variance (ANOVA) for continuous parameters.

Time-to-event analysis was performed with the Kaplan–Meier curve. When patients were lost to follow-up, the last date of outpatient control was taken to calculate the duration of thiopurine therapy following guidelines of intention-to-treat.

We used Cox regression analysis to evaluate risk factors of adverse event. Factors analyzed by univariate analysis with *P* < 0.1 were included in multivariate Cox regression. Time-to-event analysis was performed with the Kaplan–Meier curve.

The SPSS 15.0 software (SPSS Inc, Chicago, IL) was used to perform all appropriate statistical analyses. For all tests, statistical significance was set at *P* < 0.05.

## RESULTS

### Study Population

After review of 574 patients whoever received the thiopurine in our IBD center, a total of 267 patients with a minimal follow-up of 6 months were evaluated. The majority (n = 241, 90.3%) had received AZA as the first thiopurine drug. Overall, from 2000 (beginning of the prospective data collection), patients were followed up prospectively for a total of 893 patient-years on AZA/6-MP. The median follow-up with thiopurines treatment was 47.2 months (range, 2.1–100.3 months). Fifty-one (19%) patients loss follow-up in this long-term follow-up cohort, and the time to events was considered to end at the last known follow-up evaluation. The characteristics of the study population are summarized in Table 1. Two hundred nineteen had their TPMT activity detected before initiation of AZA/6-MP. The baseline TPMT activity was 13.2 ± 5.0 U/mL red blood cell (RBC) with unimodal, normal distribution (*P* = 0.79). Six patients (2.7%) had a TPMT activity of <5 U/mL RBC.

**TABLE 1:**
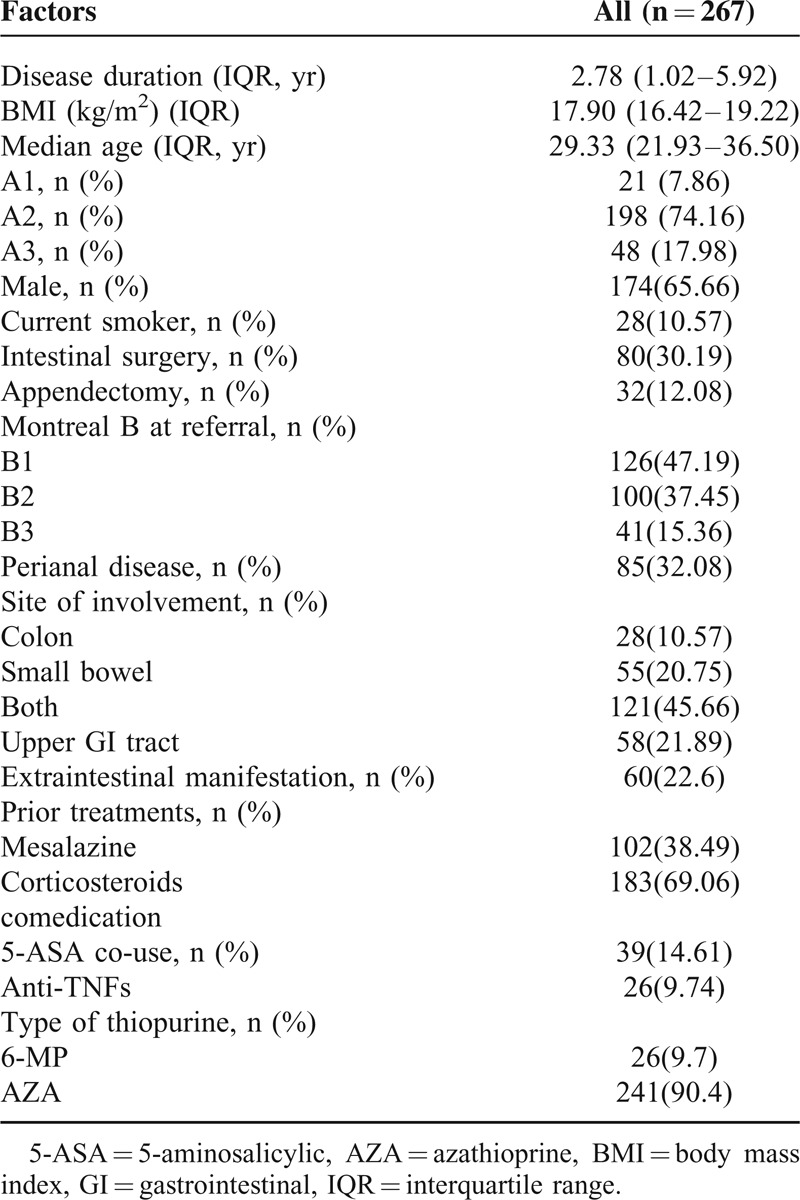
Characteristics of the Study Population

**FIGURE 1:**
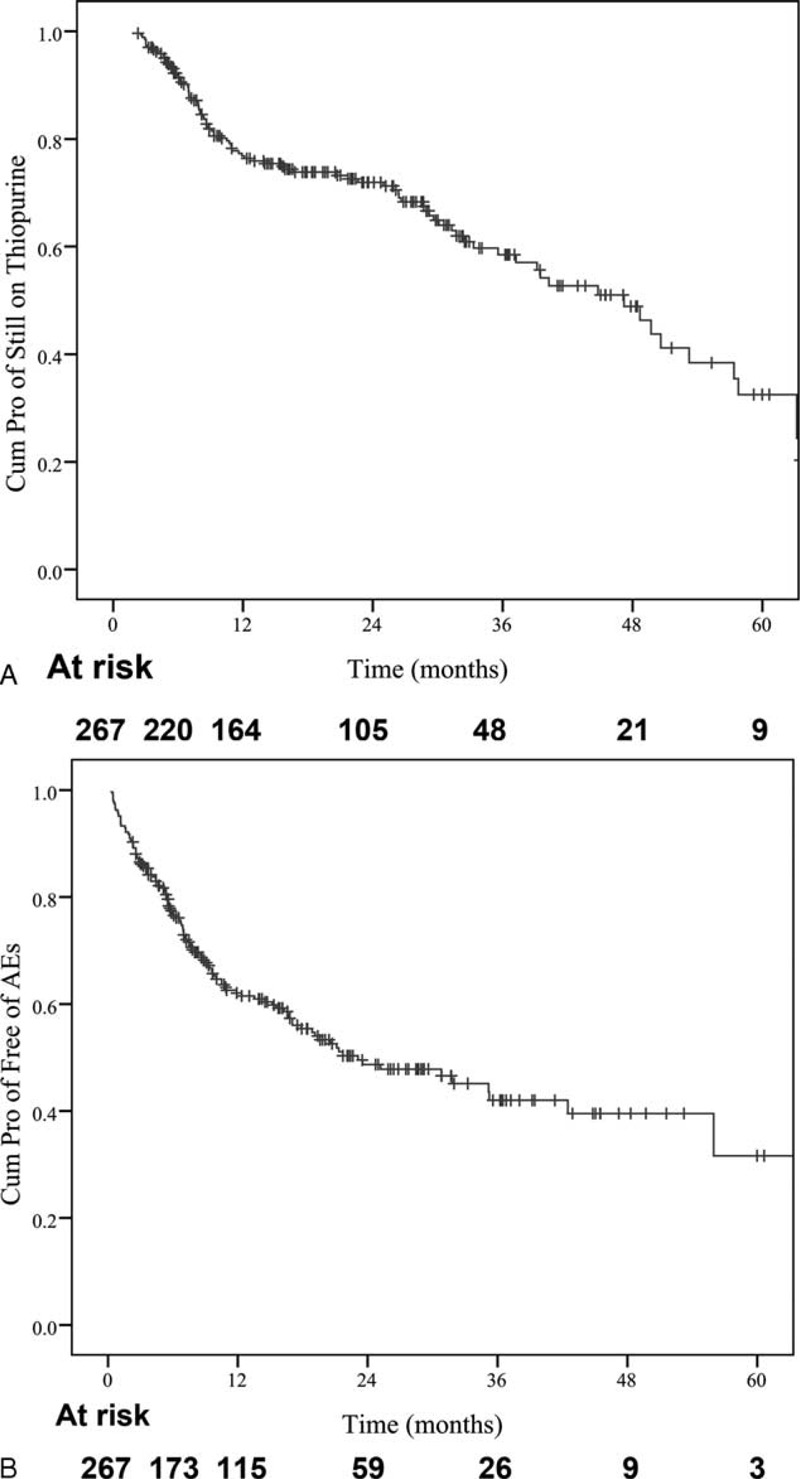
Kaplan–Meier plot showing (A) maintaining on thiopurine therapy; (B) AEs-free survival.

The percentage of patients still on thiopurines at 12, 24, 36, 48, and 60 months was 73%, 69%, 63%, 51%, and 42%, respectively (Figure 1). The median dosing of thiopurines was 1.8 mg/kg per day (IQR 1.4–2 mg/kg/d) for AZA, and 0.8 mg/kg per day (IQR 0.4–1.2 mg/kg/d) for 6-MP, with 6-TGN concentrations ranging from 172 to 309 pmol/8 × 10^8^ RBCs (median 262 pmol/8 × 10^8^ RBCs), within the target therapeutic window.

### AEs

**FIGURE 2:**
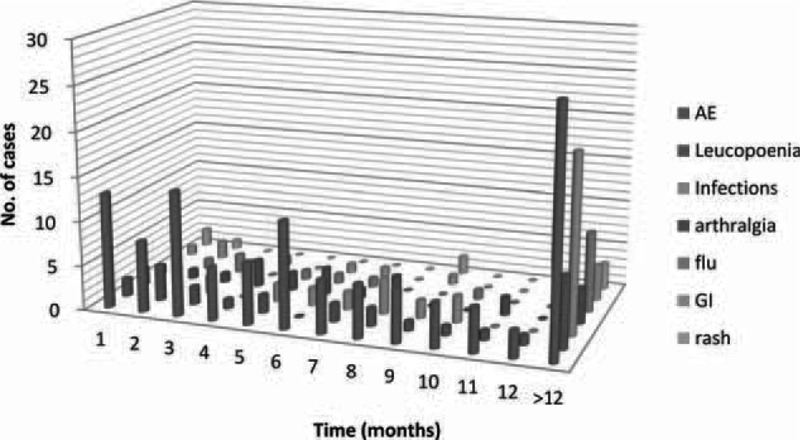
The majority of adverse events (93/143, 65%) occurred during the initial 12 mo of thiopurine therapy. Adverse events that occurred later (more than 1 yr of thiopurine therapy) consisted mainly of infections (20 of 29 cases).

Table 2 shows AEs not related to CD. Intolerance to AZA/6-MP was frequent, AEs occurred at a median of 7.4 months (IQR, 3.7–15.3 months) after starting treatment (Figure 1). The majority of adverse events (93/143, 65%) occurred during the initial 12 months of thiopurine therapy (Figure 2). The cumulative AEs-free survival was 76 ± 2.7%, 62 ± 3.2%, 48.7 ± 3.7%, 42 ± 4.3%, 39.5 ± 4.7%, 31.6 ± 8% at 6, 12, 24, 36, 48, and 60 months, respectively. The median time of AEs-free survival was 23 months (IQR, 12.6–33.6 months).

**TABLE 2:**
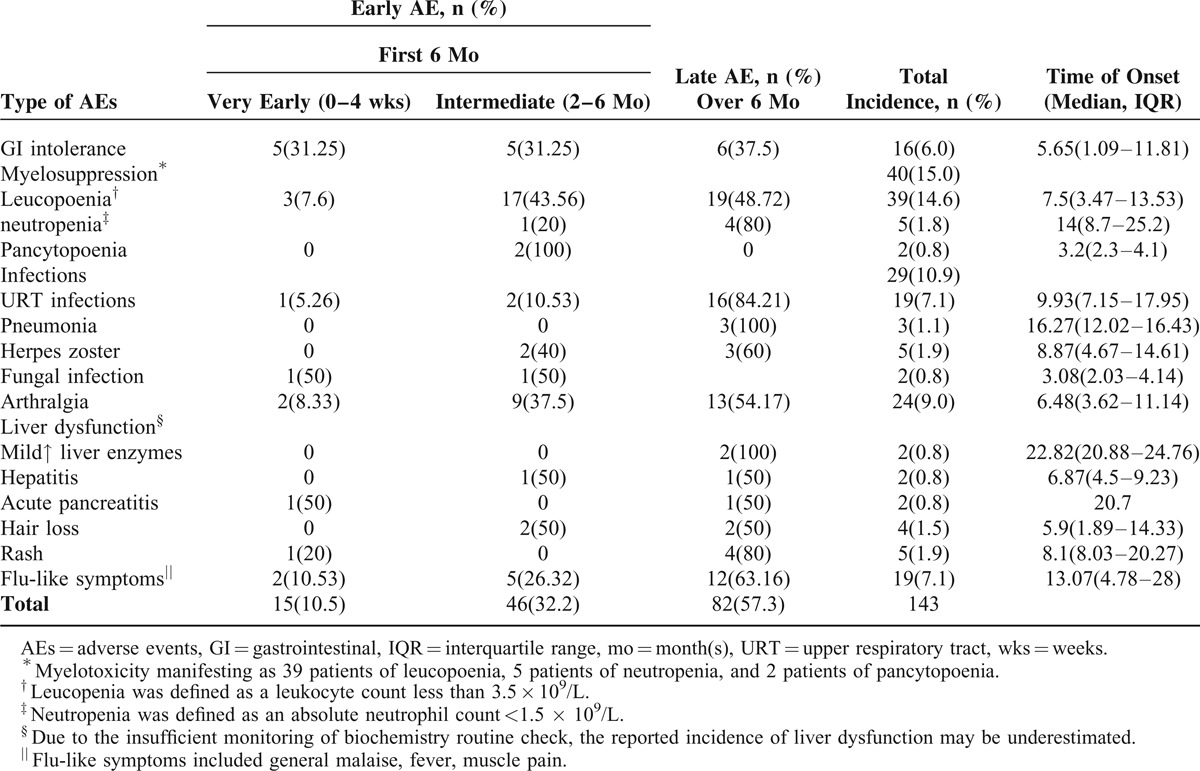
Time of Onset of the Adverse Events From the Initiation of Thiopurines

### Serious AEs

The most serious are myelosuppression, hepatotoxicity, and pancreatitis. Other clinically significant AEs are infectious occurring after drug administration, which required temporary lowering or discontinuation of the dosage. No patient died due to severe complication related to AZA/6-MP toxicity and no malignancies were observed.

### Myelosuppression

#### Leucopenia

Leucopenia occurred in 39 of 267 (14.6%) patients at a mean of 7.5 months (IQR, 3.5–13.5 months) after initiation of thiopurines (Table 2). This hematologic side-effects were managed successfully either with brief lowering of the dose (n = 10) or temporary discontinuation (n = 24) of AZA/6-MP. Twenty-two (91.7%) patients restarted AZA/6-MP, 13 (59.1%) of them had same AEs again (Table 3).

**TABLE 3:**
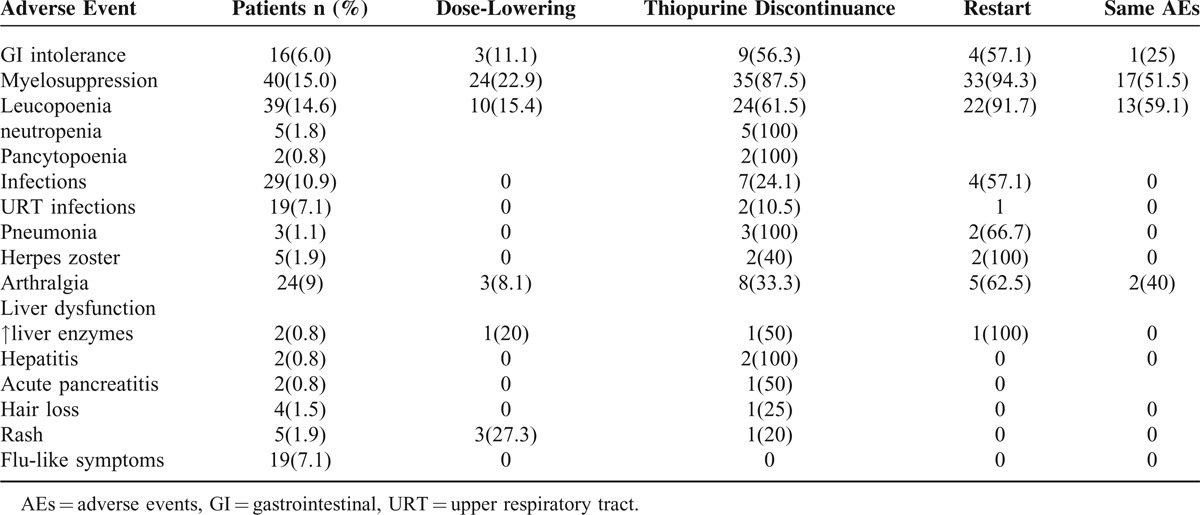
Frequency of Discontinuation of Thiopurines Due to AEs and Safety of Restarting the Treatment in Those With Previous AEs

#### Pancytopenia

Two (0.8%) patients developed pancytopenia (including neutropenia) within a median of 3.2 months (IQR, 2.3–4.1 months). Neutropenia happened in 5 (1.8%) patients within a median of 14 months (IQR, 8.7–25.2 months).

### Drug-Associated Abnormal Liver Function

Mildly abnormal liver function tests were seen in 2 patients (0.75%) within a median time of 22.8 months (IQR, 20.9–24.8), another 2 patients (0.75%) developed hepatitis within 6.9 months (IQR, 4.5–9.2) after the initiation of thiopurine within (Table 2). The liver enzyme abnormalities resolved after the reduction of thiopurines dose in all cases.

### Infectious Complications

Infectious complications occurred in 29 (10.9%) patients. AEs that occurred later (more than 1 year of thiopurine therapy) consisted mainly of infections (20 of 29 cases, 69%). The most common infections were recurrent upper respiratory tract (URT) infections (19, 8.41%). Three patients (1.12%) developed mycoplasma pneumonia and 2 patients (0.75%) developed fungal infection while taking thiopurines (Table 2). Considering the 3 patients developed pneumonia, 1 was on combination with anti-TNFs, and 1 was combined with antibodies against tumor necrosis factor (anti-TNFs) and steroids. Two of the 5 patients who developed herpes zoster were on concomitant steroids in addition to thiopurines. Both were successfully managed with oral antibiotics along with temporary discontinuation of thiopurines therapy. Herpes zoster involving the trunk was seen in 5 patients (2.21%) and both recovered regardless of thiopurines having been continued. None of the patients died due to infections.

### Minor Side Effects

*Minor* AEs were represented by arthralgia (n = 24, 9%), gastrointestinal (GI) intolerance (nausea, vomiting) in 16 patients (6%), flu-like symptoms (n = 19), rash (n = 5), and pancreatic hyperenzymemia without clinical signs of pancreatitis in 2 patients (0.8%) with a favorable nonsurgical outcome (Table 2). The digestive intolerance was managed successfully either with brief lowering of the dose (n = 3) or with temporary discontinuation (n = 9) of AZA/6-MP. Four patients restarted AZA/6-MP, 1 (25%) of them had same AEs again (Table 3).

### Pregnancy

Sixteen out of 93 female CD patients (17.2%) became pregnant while receiving thiopurines and 6 of them discontinued the drug after balancing the risk/benefit ratio. One of these pregnancies was extrauterine; no adverse outcomes were observed.

### Predictors of AEs

Predictive factors of the development of the most frequent AEs were demonstrated in Table 4. By multivariate analysis, only a higher 6-TGN concentration (>384 pmol/8 × 10^8^ RBCs) was significantly associated with the increased overall AEs. The concomitant use of 5-aminosalicylic acid (5-ASA), dosage, type of thiopurine, the concentration of 6-TGNs, the baseline Hct, Hb, the sex, and the TPMT were included into Cox regression analysis. Multivariate logistic regression identified a lower baseline hemoglobin (< 108 g/L) when initiate thiopurines treatment (HR, 0.34; 95%CI 0.18–0.67) and the concomitant use of 5-ASA (HR, 3.05; 95 % CI 1.44–8.76) independent predictive of leucopoenia. Infection was significantly associated with the use of 6-MP (HR, 5.03; 95% CI 1.35–18.71). No independent predictive factors were identified for the incidence of arthralgia.

**TABLE 4:**
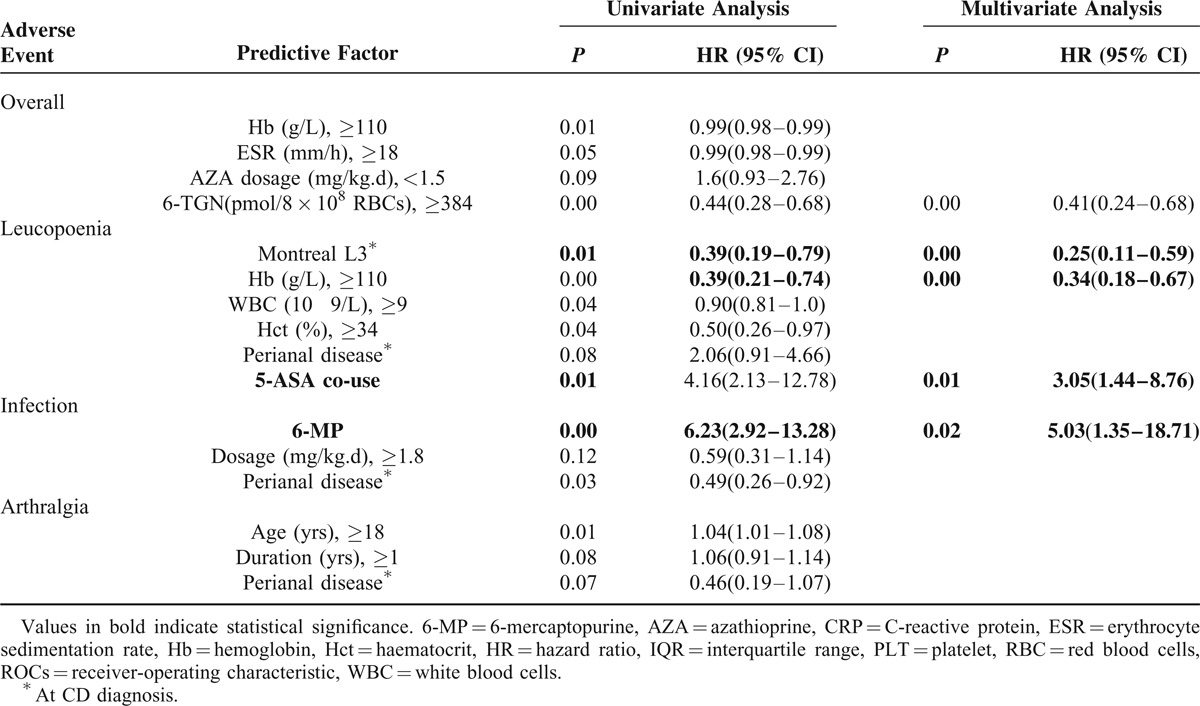
Predictive Factors Associated With the Development of Adverse Events

### Discontinuation of Therapy

In the whole study population, drug withdrawal from the study occurred in 76 of 267 patients (28.44%). The reasons for thiopurine therapy discontinuation at any time were: 39 (14.6%) patients because of AEs, 19 (6.9%) patients because of inefficacy (12 patients relapsed, 7 patients underwent operation), and 6 (2.2%) patients because of reaching the 5-year therapy limit.^17^ Seven (2.5%) patients stopped with arbitrary reason (safety concerns, pregnancy, poor compliance, etc., Table 5).

**TABLE 5:**
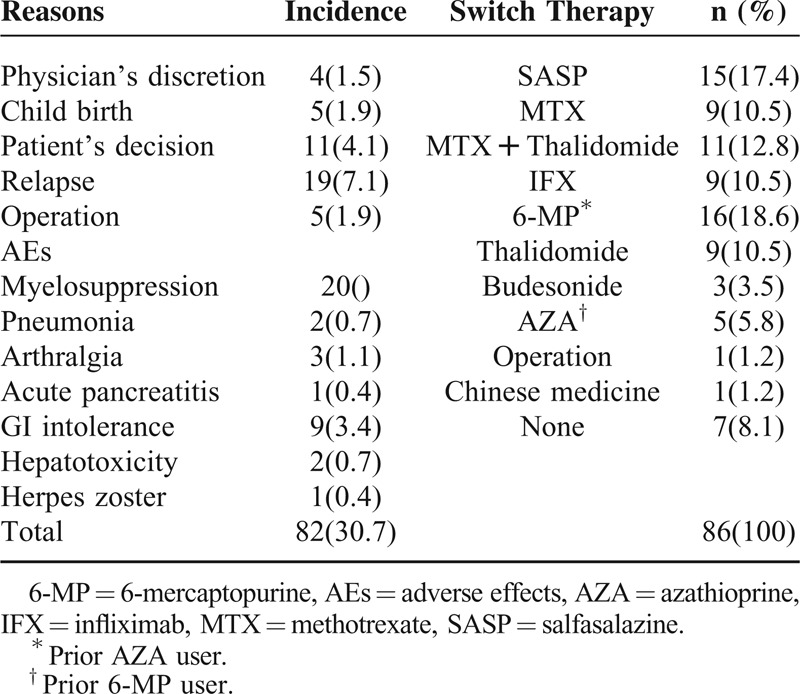
Discontinuation of Thiopurines in CD Patients Along With Reasons to Withdraw

### Predictors of Discontinuation of Therapy

Lower body mass index (BMI) (<18 kg/m^2^) (HR, 1.59; 95% CI, 1.04–2.46), the presence of extraintestinal manifestation at CD diagnosis (HR, 1.85; 95% CI, 1.18–2.88), and the incidence of leucopenia (HR, 1.76; 95% CI, 1.05–2.97) were associated with increased risks of the cessation of thiopurine treatment by multivariate analysis (Table 6).

**TABLE 6:**
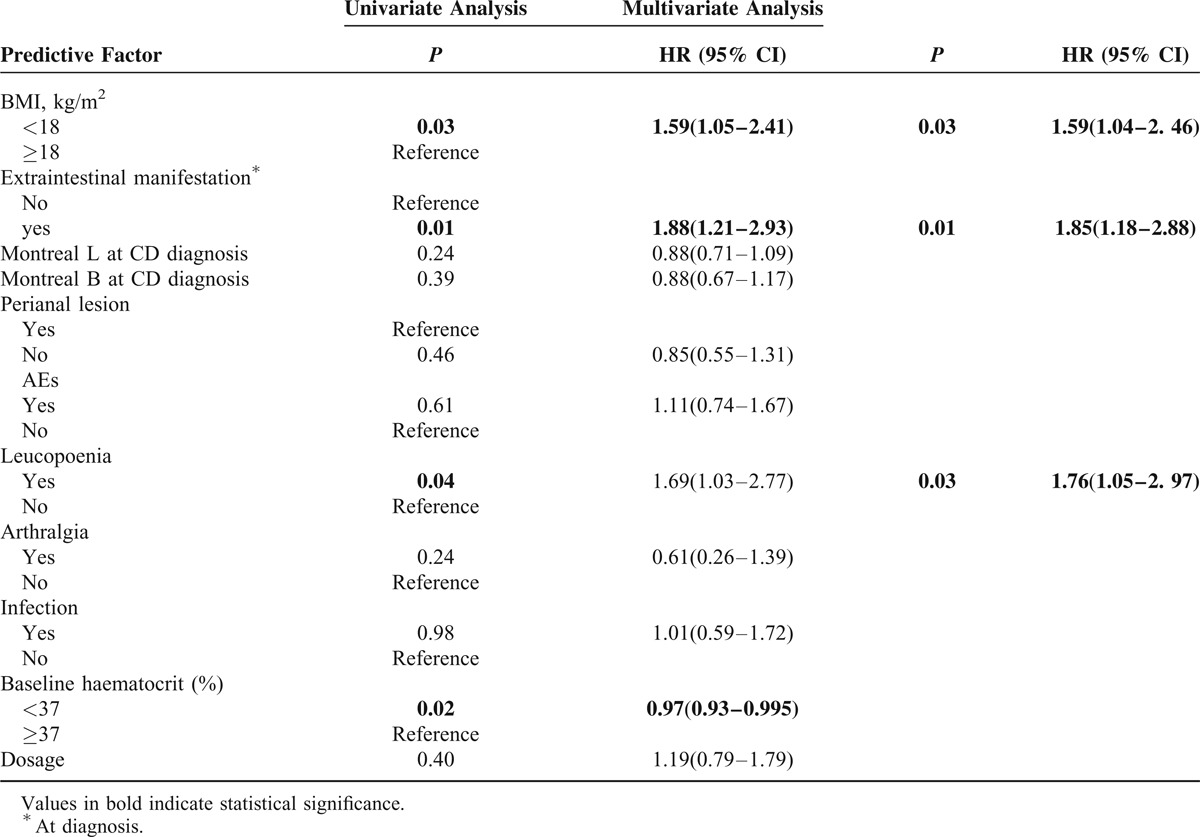
Predictive Factors Associated With the Discontinuation of Therapy

### Switch Therapy

We also evaluated the efficacy and safety of converting azathioprine into other immunosuppressants (ie, anti-TNFs, 6-MP, thalidomide, and/or methotrexate). AZA was switched successfully to 6-MP in 16 patients who terminated therapy due to AEs including myelosuppression (n = 11), arthralgia (n = 2), or GI intolerance (n = 2). Both thalidomide (n = 9) and methotrexate (n = 9) were well tolerated in patients refractory to thiopurine. The 12 relapsed patients were managed successfully either by combined therapy with methotrexate and thalidomide (n = 6) or by the introduction of anti-TNFs (n = 6, Table 5). Furthermore, 2 patients with a low 6-TGN concentration terminated therapy due to intolerance after adding allopurinol. None of the whole population tried the use of thioguanine.

## DISCUSSION

In this study, we assessed the safety profiles of thiopurines in a large group of Chinese Han patients affected by CD. In our sample, AZA was largely preferred to 6-MP, in accordance with the European practice.^18^ The cumulative incidence of AEs was 26%, with an annual risk of 7% per patient-year of treatment which is consistent with the Chaparro et al study.^6^ The spectrum of AEs associated with thiopurines is detailed in Table 1. The prevalence of the single side effects varies among the different studies, partly due to the use of nonunivocal definitions. The rates of myelotoxicity (15%), infections (11%), arthralgia (9%), flu-like symptoms (7%), GI reactions (6%) observed in the present study do not significantly differ from literature data.^14,19–23^ The reported incidence of liver dysfunction (1.50%) may be underestimated when compared with the reported incidence of 4% ^6^ due to the insufficient monitoring of biochemistry routine check, and a relatively lower median dosage of thiopurine, as hepatotoxicity is usually considered dose-dependent reactions. In our sample, no drug-related mortality was observed.

Isolated leucopenia is the most common hematological side effect of thiopurines, appearing in 14.23% of the patients, a figure that is similar to the cumulative incidence of myelotoxicity estimated in a recent systematic review,^24^ although higher than the reported incidence of 4.1% by Chaparro et al.^6^ In the present study, the median time from thiopurines commencement to the onset of leucopenia was 7.5 months (IQR, 3.5–13.5 months). Myelosuppression may occur at any time during therapy according to our study, continued monitoring of blood cell counts, at regular intervals, throughout therapy is necessary.

Recent surveys have pointed out the increased risk of infections in association with immunosuppressants (corticosteroids, AZA/6-MP and infliximab).^25^ In our cohort, infections occurred in 29 (10.9%) patients, and mainly occurred later (more than 1 year of thiopurine therapy) (20 of 29 cases, 69%, Figure 2). The development of herpes zoster was observed in 5 patients (1.87%). The annual incidence of shingles is approximately 12%, and our findings were similar. Serious bacterial (pneumonia, n = 3), fungal infection (n = 2), or viral infections were scarcely observed during 5-year follow-up on AZA/ 6-MP therapy. This finding may be at least partially justified by the fact that severe or continued leucopenia was uncommon in the current cohort.

More than the infectious risk, the fear of malignancy is a major reticence to prolonged use of AZA/ 6-MP. In our sample, no drug-related lymphoma was observed, only 1 female developed breast cancer which probably should not attribute to the thiopurine use. As a consequence, in our series thiopurines do not appear to increase the risk of developing neoplasia, in agreement with a previously published meta-analysis showing no difference in the incidence of any kind of malignancy in IBD patients who received immunosuppressants compared with those who did not.^26^

There is significant uncertainty around predicting which patients will respond to therapy with thiopurines. AEs can occur at any time and are frequently unpredictable.^6^ In the current study, several predictors both for AEs have been identified. By multivariate analysis, only a higher 6-TGN concentration (>384 pmol/8 × 10^8^ RBCs) was significantly associated with the increased overall AEs. Multivariate logistic regression showed independent predictive factors of leucopenia on a lower baseline hemoglobin (<108 g/L) while initiating thiopurines treatment (HR, 0.34), and the concomitant use of 5-ASA (HR, 3.05). Infection was significantly associated with the use of 6-MP (HR, 5.03). The concomitant use of 5-ASA which may be correlated to the increase in 6-TGN has been reported previously.^16^ However, there was no correlation between myelosuppression and the mean concentration of TPMT as we have demonstrated before.^15,16^ On the contrary, we previously confirmed that the hypoxanthine guanine phosphoribosyltransferase (HPRT) activity is related to 6-TGNs concentrations and thiopurine-induced leucopenia in the treatment of IBD.^15^ Consequently, the European Crohn's and Colitis Organisation (ECCO) do not recommend the routine measurement of TPMT activity or genotype before initiating thiopurine therapy.^27,28^ Chaparro et al^6^ identified female and a diagnosis of CD was associated with an increased risk of overall AEs. However, no sex difference exists considering the incidence of AEs in our study.

The discontinuation rate and reasons for cessation of thiopurine therapy were additionally assessed. Overall, 26% of CD patients discontinued therapy in our study, with a discontinuation rate due to side effects of 14.61%, followed by refractoriness (4.49%) or patient's request (2.62%). In previous studies, thiopurines discontinuation rate due to side effects varies from 5% to 6% ^14,21^ to up to 30%.^29,30^ Regarding the risk factors, our study demonstrated that a lower BMI (<18 kg/m^2^), the presence of extraintestinal manifestation at CD diagnosis, and the incidence of leucopenia independently predict a higher probability of drug withdrawal by multivariate analysis.

Alternative management of patients in whom thiopurine drugs have to be discontinued due to AEs is unclear. An approach may be to use mercaptopurine when an AE has developed to AZA (or vice versa). In our cohort, 16 AZA users successfully switched to 6-MP. The most favorable results with this strategy were observed in patients with leucopenia (n = 9) arthralgias/myalgias (n = 4), or GI intolerance (n = 3) as the primary reason for discontinuing AZA.

This study had several limitations. First, the retrospective design could induce a bias of selection and a bias of gathering information. However, most of the data (from 2003 to 2013) were collected prospectively and made these biases minimal. Second, the relatively high proportion of patients lost to follow-up evaluation (19%), in part related to the duration of the study, up to 13 years, should also be taken into account. Third, tertiary-center referral bias was also likely to have influenced the outcomes considering the data source from a referral teaching hospital. Further well-designed RCT studies addressing the above issues are required. Last but not least, the thiopurine dose was chosen based on the 6-TGNs concentrations with a target therapeutic window of 250 to 400 pmol/8 × 10^8^ Erythrocyte. Therefore, the incidence of AEs found in the present study was probably lower than if this strategy was not performed.

In conclusion, despite the fact that thiopurine dose was chosen based on the 6-TGNs concentration, a relatively high incidence of AEs was found. Higher 6-TGN concentration was significantly associated with the increased AEs. Specifically, we identified 2 factors independently associated with a subsequent occurrence of leucopenia: lower baseline hemoglobin and the concomitant use of 5-ASA. Moreover, lower BMI, the presence of extraintestinal manifestation at diagnosis, and the incidence of leucopenia were associated with the cessation of thiopurine treatment.
